# Development of Edible Carbohydrate–Protein Sports Gels to Optimize the Muscle Glycogen Re-Synthesis

**DOI:** 10.3390/gels11050341

**Published:** 2025-05-02

**Authors:** Vishal Verma, Vishal Gill, Avinash Kumar, Shailendra Pratap Singh

**Affiliations:** Department of Sports Bio-Sciences, School of Sports Sciences, Central University of Rajasthan, Ajmer 305817, India; vishal_131@yahoo.com (V.V.); vishalgill4699@gmail.com (V.G.); avinash.kumar.ap@gmail.com (A.K.)

**Keywords:** hemp seed (*Cannabis sativa* L.), sports gel, protein, functional food, muscle recovery, performance, athlete, Bengal gram dal (*Cicer arietinum* L.)

## Abstract

This study was aimed at providing athletes a solution to replenish the muscle glycogen re-synthesis at an optimal rate with hemp seeds as a natural protein source and Bengal gram dal and its use for the preparation of gel. The gel contains the richest source of energy, and it is an effective way to provide energy and nutrients to athletes. The gel was prepared in three variations with different hemp seed concentrations. We then analyzed the gel for pH and macronutrient composition. The sensory characteristics were analyzed for seven parameters, including appearance, taste, color, texture, aroma, consistency, and acceptability, using a hedonic scale on 25 panelists. A sensory analysis showed that sample A received an overall acceptability score of 7.16 ± 0.99 from the sensory panel. The shelf life was observed at the recommended temperature of 4 degrees Celsius, which was 12 days. The best formulation was sample B with 38 g of hemp seeds, which showed better taste, color, aroma, and acceptability and a lower average pH value (6.68 ± 1.44, 6.56 ± 1.29, 7.6 ± 1.16, 7 ± 1.26, and 5.822 ± 0.0183, respectively). Sample B contained 30.8 g of protein, 16.09 g of carbohydrates, 8.4 g of fat, and 263.16 kcal of energy per 100 g. The resulting ratio of carbohydrates to protein is optimal for use as a high-protein post-workout meal. Hence, it can be considered a post-workout supplement.

## 1. Introduction

Muscle glycogen is important for endurance as well as strength exercise because it serves as a major source of carbohydrates during exercise. Ivy (1991) [1], in his research, found that when athletes have greater muscle glycogen storage, the time to exhaustion increases. They also mentioned that muscle glycogen replenishment is a prerequisite for achieving optimal performance [1]. The addition of protein in the carbohydrate supplement reduces the muscle damage markers and helps athletes to effectively recover from workout-induced damage. Saunders et al. found in their research that the group of athletes who ingest carbohydrates with protein gel prevent increasing muscle damage and prolong endurance performance [2]. One of the main ingredients used as a source of protein in the formulation of this gel is hemp seed (*Cannabis sativa* L.).

Hemp seed is a great alternative protein source for vegetarians, and having a high nutritional value, it is referred to as a magnificent source of nourishment, especially essential amino acids and unsaturated fatty acids [3]. Hemp typically contains 25–35% of fat, 20–25% of protein, and 20–30% of carbohydrates [4]. It also contains great antioxidant potential as well as phenolic compounds, tocopherols, carotenoids, and phytosterols [5]. The hemp seeds’ Omega-6-to-Omega-3 ratio ranges from 3.2 to 5.1 [6]. The optimal ratio is 1:1 to 2:1. Industrial hemp is also a rich source of Linolenic Acid (18:2, LA) (most representative) (precursor of Omega-6) and Alpha Linolenic Acid (18:3, ALA) (precursor of Omega-3). Additionally, γ-Linolenic Acid (18:3, GLA) and Stearidonic Acid (18:4, SDA) are present in hemp [4]. Industrial hemp is distinct from recreational hemp due to two major phytochemical compounds known as delta-9-tetrahydrocannabinol (THC), which is a psychoactive and toxicant compound of the plant, and second, as Cannabidiol (CBD), which is non-psychoactive. THC and CBD compounds tend to be higher in recreational hemp, based on these compounds’ content. Chemotype I is high in THC and low in CBD and is used for recreational purposes. Chemotype II has a balanced CBD/THC ratio and is used for medicinal purposes. Chemotype III is high in CBD and low in THC and is used in industrial applications [4]. Hemp seeds contain two main proteins: edestin (67–75% with a molecular weight of 31.5 KDa) and albumin (25–37% with a molecular weight of 58.1 KDa), and a minor percentage is conglycinin (up to 5%) [7,8]. Hemp protein is always referred to as complete protein because it has high levels of arginine (15.52 g/100 g), phenylalanine + tyrosine (9.63 g/100 g), methionine + cysteine (5.49 g/100 g), leucine + isoleucine (5.21 g/100 g), and valine (4.53 g/100 g). These amino acid levels are used to determine the nutritional quality of protein. Tryptophan is the limiting amino acid (0.22 g/100 g), while it has moderate levels of lysine (2.50 g/100 g) and threonine (3.29 g/100 g) [7].

The addition of hemp seeds as a component in sports gel can increase the amount of protein in the gel with a complete ratio of amino acids along with different properties of hemp seeds persisting, like antimicrobial, antioxidant, antifungal, immuno-modulatory, neuroprotective, cardio-protective, premenstrual syndrome and menopause, anticancer and anti-inflammatory properties [9,10].

Bengal gram dal (*Cicer arietinum* L.) was added to increase the protein content of the gel, as it is a rich source of protein and nutrients such as folate, beta-carotene, and health-promoting fatty acids. It reduces the risk of chronic illnesses [11]. Honey was added to increase the phytochemical, anti-inflammatory, anti-microbial, and antioxidant (flavonoids and polyphenols) properties [12]. Honey reduces physical injury by improving Hemoglobin and Creatine kinase content and also reduces the blood urea nitrogen. Muscle fibers are protected from damage, and at the same time, ATP is replenished during and after exercise and delayed fatigue [13].

Pulses largely consumed in India are indeed a reliable and affordable source of food with dense energy and protein, minerals, and vitamins. The Bengal gram dal has a quality of dryness and softness. For clinical purposes, Bengal gram dal is very useful for the treatment of children’s kwashiorkor, impotence, early ejaculation, liver hardness, underweight, etc. Bengal gram flour is a highly potent purifying agent [14]. The chickpea (*Cicer arietinum* L.) is found to be the world’s third most consumed legume [15,16]. Bengal gram contains 330.48 kcal of energy, 46.72 g of carbohydrates, 21.55 g of protein, and 5.31 g of fat, respectively [17]. It has been found that this chickpea is a good source of protein and carbohydrates, which preserves better quality compared to other legumes like green gram, black gram, and pigeon pea. It also supplies vitamins like thiamine and niacin and minerals such as calcium, magnesium, zinc, potassium, iron, and phosphorus [18]. Bengal gram dal (*Cicer arietinum*) is high in protein, which makes this dal the best replacement for a non-vegetable protein source for vegetarian people. It is also a good source of fiber, vitamins, and minerals, which makes it good for the digestive system and weight maintenance [19].

Honey is a natural sweetener and is the only substance produced by insects that is edible. Bees produce honey from the nectar of blossoms; bees secrete the honey from the living parts of plants, and they keep the material and mix it with specific substances. Then, they leave the honey in the honeycomb to ripen and mature. Honey includes carbohydrates that almost cover 82% of the honey, and proteins vary between 0.5 and 0.2% in terms of enzymes such as glucose oxidase, invertase, catalase, diastase, and 18 Free AA. Out of all of them, the most abundant was found to be proline. Other than sugar and protein, honey also contains other compounds such as minerals, vitamins, enzymes, and a wide range of polyphenols [20,21]. Honey’s nutrients depend on factors like bee type, floral source, environment, and processing [21]. Küçük et al. (2005) checked the honey composition of three honey samples and found that the principal carbohydrate was fructose 28–40%, glucose 20–35%, and disaccharide and tri-saccharide concentration was around 5 to 1%, respectively [22]. Maltose, turanose, sucrose, and nigerose were the most identified disaccharides in the honey [21]. Honey is also found to have anti-inflammatory properties, and it increases the synthesis of cyclooxygenase-2 and prostaglandin, two inflammatory mediators [21].

Skeletal muscles have an endurance exercise capacity, which is associated with their glycogen content, and when muscle glycogen content drops due to prolonged exercise, muscles feel exhausted and fatigued [23].

In the cytosol, two enzymes facilitate the glycogen breakdown, or glycogenolysis, by glycogen phosphorylase and the glycogen de-branching enzyme, which both help in releasing glucose 1-phosphate and untangle the branch points of glycogen, respectively. In the lysosomes, α-glucosidase catalyzes the breakdown of glycogen. Free glucose comes out of the cell with the help of the glucose 6-phosphatase system, which dephosphorylates glucose 6-phosphate to glucose [24]. After all of these mechanisms, or immediately after a workout, muscle glycogen, which was fueling the workout, is depleted and triggers the mechanism of rapid glycogenesis or glycogen replenishment. When athletes ingest carbohydrates soon after a workout or training, the insulin released from the pancreas in response to the glucose increment in blood, insulin sensitivity in muscle cells initiates the glucose uptake from blood; this increases the glucose synthase activity [25,26], and this response stays for as long as 48 h [27,28]. Hemp seeds combined with aerobic physical activity show significant results in improving weight, BMI, fat mass, HDL, catalase, and brain-derived neurotrophic factors and improve health parameters like cardiovascular conditions, free radical stress, and insulin resistance [29].

CBD found in hemp seeds (*Cannabis sativa* L.) contains anticonvulsant properties and has therapeutic effects for athletes, too. WADA no longer recognizes CBD as a prohibited substance, and it appears safe for athletes. High-intensity exercise initiates an inflammatory response, and too much inflammation causes muscle soreness and delayed recovery. CBD was found to modulate this inflammatory response and attenuate immune cell accumulation, stimulate the production of Interleukin-4, -10, and inhibit the production of Interleukin-1β, -6, -8, tumor necrosis factor (TNF-α), and reactive oxygen species [30]. Consuming CBD during endurance running raises oxygen utilization and enjoyment levels [31]. Preclinical research has shown that CBD has anti-inflammatory, anti-apoptosis, and antioxidative stress properties that may help prevent cardiac damage during intense exercise [32]. CBD seems to have all of these properties and possibly recovery-mediating qualities for athletes, but more scientific evidence is required to establish these effects [33]. According to preclinical research, CBD’s anti-inflammatory, analgesic, anxiolytic, neuroprotective, and sleep–wake cycle-influencing qualities may make it beneficial for athletes. Unfortunately, there are few studies on these effects during exercise; thus, its application is still in the premature stage [34].

Honey is mainly a carbohydrate source and, along with it, consists of minerals and vitamins, which are seen as an ability to improve exercise performance and general health. Honey constituents on their own may enhance aerobic exercise performance like cycling and running. At the same time, honey consists of flavonoid compounds that act as antioxidants and have ergogenic properties, and incorporating honey with exercise can benefit bone health, boost immune system function and reproductive hormones, and improve inflammatory responses [35,36]. In comparison to other carbohydrate sources, consuming honey has shown comparable effects on exercise performance, feelings of fatigue, blood glucose levels, and immune reactions when ingested just before or during physical activity, although some beneficial effects have been noted. When consumed regularly over several weeks, honey may reduce various immune disturbances commonly linked to a regimen of moderate-to-high-intensity exercise [37].

**Figure 1:**
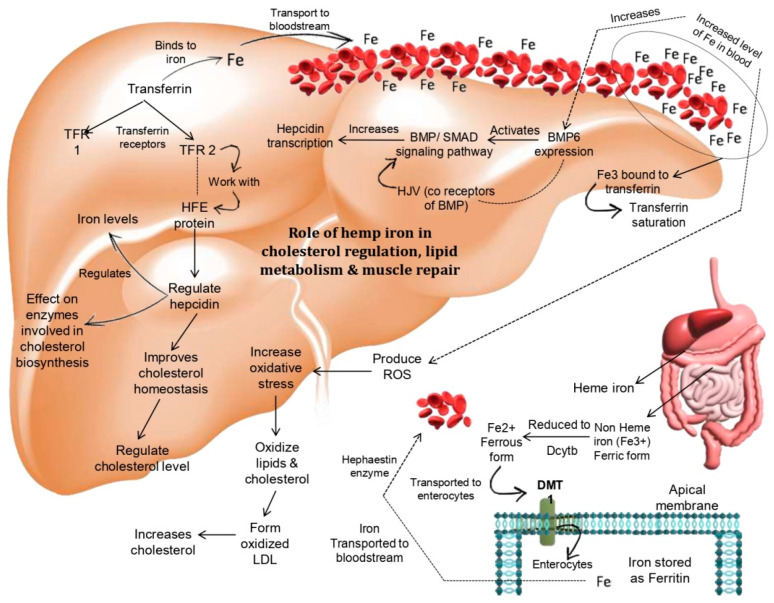
Hemp iron plays a role in muscle regeneration, lipid metabolism, and cholesterol management. Iron from hemp seeds that is coupled to transferrin and carried into the bloodstream raises BMP6 expression, which in turn triggers the BMP/SMAD signaling pathway, which ultimately triggers the transcription of hepcidin. Among the transferrin receptors, TFR1 and TFR2, which are coupled to iron, TFR2 regulates hepcidin by working with the HFE protein. This enhances cholesterol homeostasis, which further controls cholesterol levels and promotes the best possible muscle health and repair. At the same time, TFR 2 influences the enzymes involved in the manufacturing of cholesterol and controls the ideal amounts of iron (by author Avinash Kumar, 2025).

This study aimed to develop a carbohydrate–protein gel from hemp seeds (*Cannabis sativa* L.), Bengal gram dal (*Cicer arietinum* L.), and honey. This study targeted the development of a sports gel that utilizes hemp seeds (*Cannabis sativa* L.) in different proportions as raw materials, and this is formulated with hydrocolloid xanthan gum. This product can be helpful for athletes, serving as an optimal ratio of carbohydrates and protein that can optimize muscle glycogen re-synthesis after exercise (Figure 1). The resulting product was a gel based on hemp seeds, Bengal gram dal, and honey, which was analyzed for nutritional content and sensory characteristics.

## 2. Results and Discussion

### 2.1. Physicochemical Characteristics 

For the pH, the results say the pH of the gel was significantly different in all variations, shown by *p*-values < 0.05 (*p* < 0.001). Further testing with Duncan’s multiple range test showed that the gel did not have any significant differences between them, shown by *p*-values < 0.05 (*p* = 0.23). Although the results show no significant differences between pHs, the mean of sample B was noted to be higher compared to both samples A and C (Table 1).

**Figure 2:**
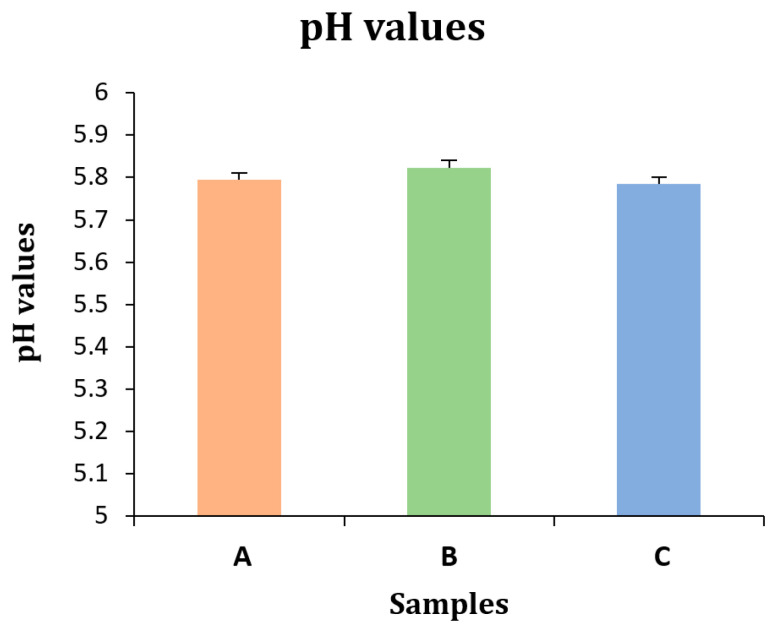
pH values of all three samples with standard deviation (by author Vishal Verma, 2025).

The lab testing of the pH of all three samples showed the highest value for sample B, while the statistical results showed no differences in pH, indicated by *p*-values < 0.001. Sample A represents gel with 32 g of hemp seeds, sample B represents gel with 38 g of hemp seeds, and sample C represents gel with 42 g of hemp seeds (Figure 2).

The solubility profile of hemp seed protein is U-shaped, with the lowest solubility occurring close to its isoelectric point (~pH 5.0). Between pHs of 4.0 and 6.0, it retains roughly 70% solubility, which makes this range perfect for smooth, protein-rich compositions like sports gels. While avoiding precipitation, maintaining a pH between 4.0 and 5.5 guarantees the best possible protein solubility and texture [7,38]. Xanthan gum, a common thickening ingredient that stays stable and viscous in acidic conditions, works well in this pH range as well. Research indicates a high synergy between hemp protein and xanthan gum, since it increases gel strength, spanning pHs of 4 to 7. In this ideal acidity range, they combine to form a stable, smooth-textured sports gel [39].

For energy content, the energy contents of all three sports gels are as follows: sample A = 262.7, sample B = 263.2, and sample C = 270.5. Sample C contains the highest energy content (Table 2). In terms of carbohydrate content, the carbohydrate content of all three sports gels is as follows: sample A = 12.73, sample B = 16.09, and sample C = 15.71. Sample B contains the highest carbohydrate content (Table 2). In terms of protein content, the protein content of all three sports gels is as follows: sample A = 30.9, sample B = 30.8, and sample C = 31.9. Sample C contains the highest protein content (Table 2). The Fat content of all three sports gels is as follows: sample A = 8.9, sample B = 8.4, and sample C = 8.9. Samples A and C were found to have the same fat content (Table 2). The Ash content of all three sports gels is as follows: sample A = 1.92, sample B = 1.77, sample C = 1.85. Sample A was found to have a high ash content (Table 2). The moisture content of all three sports gels is as follows: sample A = 44.65, sample B = 42.94, and sample C = 41.64. Sample A was found to have high moisture content (Table 2). All testing was performed in the FICCI Research and Analysis Centre, New Delhi.

### 2.2. Sensory Characteristics of Sports Gel

(a) Appearance

**Figure 3:**
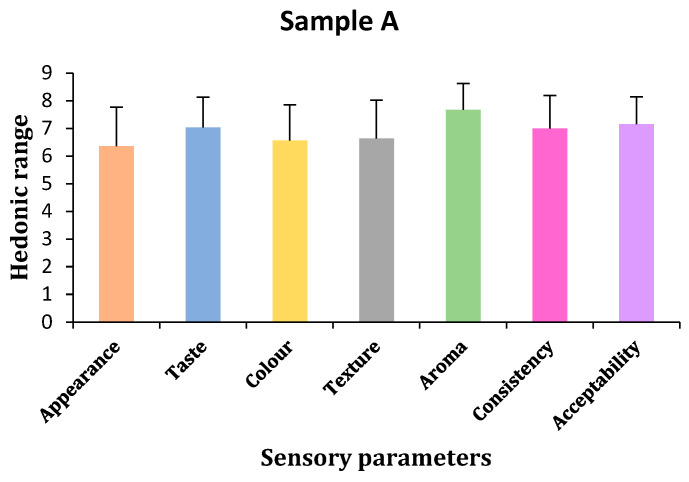
Sensory parameters of sample A (by author Vishal Verma, 2025).

**Figure 4:**
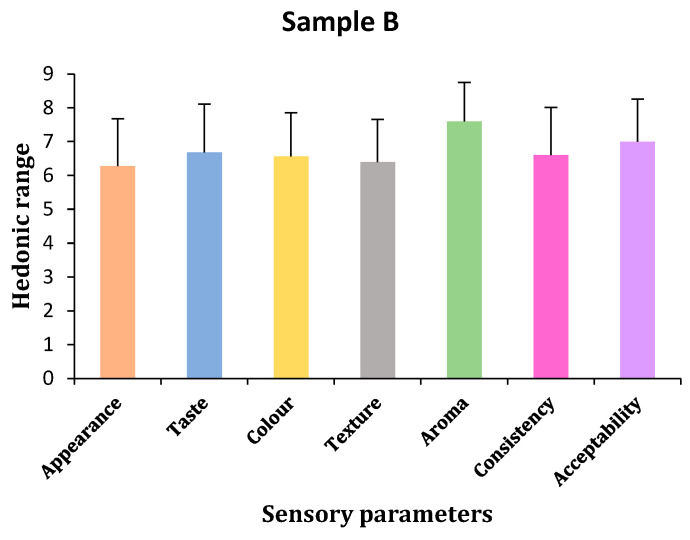
Sensory parameters of sample B (by author Vishal Verma, 2025).

**Figure 5:**
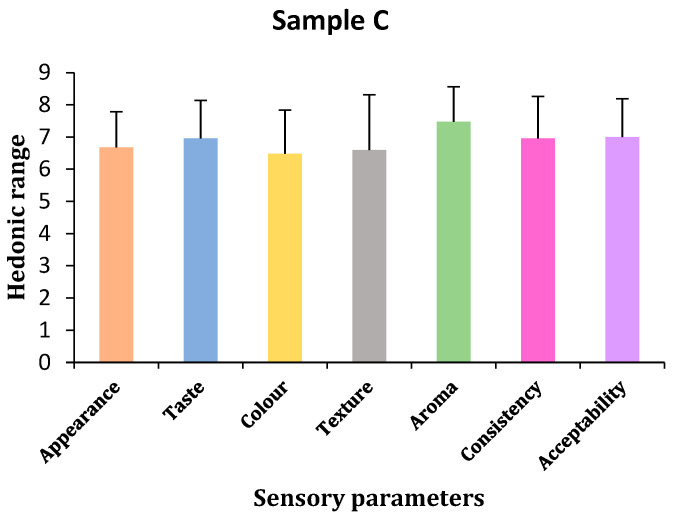
Sensory parameters of sample C (by author Vishal Verma, 2025).

Hedonic test results showed that panelists chose sample C for appearances, followed by samples A and B. However, statistical results showed that there were no significant differences in all samples, shown by *p*-values < 0.05 (*p* = 0.611). Bonferroni tests confirmed no differences between samples A and B and slight differences between samples B and C (Table 3 and Figure 3, Figure 4 and Figure 5).

(b) Taste

The panelists of the hedonic test chose sample A for taste, followed by sample C and sample B, with sample C being slightly closer to A. Statistical analysis showed no significant differences in taste among the three samples (*p* = 0.729). Bonferroni tests confirmed no taste differences between samples C and both A and B, but there were slight differences in taste between samples A and B (Table 3 and Figure 3, Figure 4 and Figure 5).

(c) Color

The panelists could not find any color difference between samples A and B based on the hedonic test findings, but they did not like sample C’s color, which had a slight but non-significant difference. According to statistical analysis, there was no change in color, shown by the *p*-value of *p* < 0.05 (*p* = 0.972). Subsequent analysis using the Bonferroni test revealed no differences between any of the samples (Table 3 and Figure 3, Figure 4 and Figure 5).

(d) Texture

According to the hedonic test results, panelists preferred sample A’s texture over samples C and B. Further testing from statistical tools showed that there was no difference in the texture of all three samples, indicated by a *p*-value < 0.05 (*p* = 0.711). Bonferroni test results also did not show any differences among the samples (Table 3 and Figure 3, Figure 4 and Figure 5).

(e) Aroma

Hedonic test results showed that the panelists liked the aroma of sample A, followed by the B and C samples. Samples A and B were similar, but C had slight differences. Statistical tools confirmed that no sample had any differences in the aroma of all three samples indicated by a *p*-value < 0.05 (*p* = 0.787). Bonferroni test results also did not show any differences among the samples (Table 3 and Figure 3, Figure 4 and Figure 5).

(f) Consistency

In terms of consistency, the hedonic test results showed that the panelists liked sample A, followed by samples C and B. Statistical tools showed no differences in the consistency of all the samples, indicated by *p* values < 0.05 (*p* = 0.685). Bonferroni test results showed that sample A had few differences in consistency compared to sample B and was similar to sample C, and samples B and C had no differences between each other (Table 3 and Figure 3, Figure 4 and Figure 5).

(g) Acceptability

Hedonic results confirmed that sample A was more acceptable than samples B and C, although samples B and C were likely acceptable to the panelists. Statistical tools showed no differences in acceptability by panelists in all three samples. Bonferroni test results also did not show any differences among the samples. All three samples have the same acceptability (Table 3 and Figure 3, Figure 4 and Figure 5).

### 2.3. Correlation Between Sensory Parameters Evaluated

**Figure 6:**
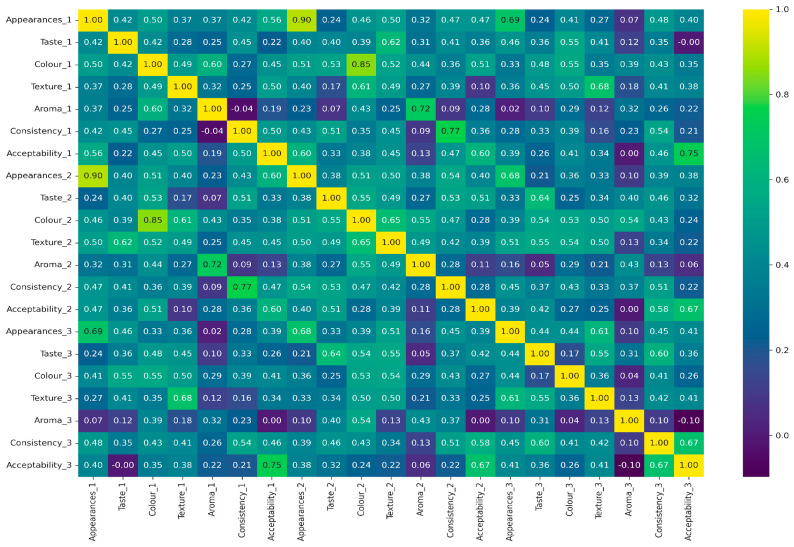
Correlation heatmap showing the relationship between sensory attributes, i.e., appearance, taste, color, texture, aroma, consistency, and acceptability, across three samples, highlighting the key drivers of overall product acceptability (by author Vishal Verma, 2025).

The relationship between the sensory attributes of all the variations of the gels is shown in Figure 6.

#### 2.3.1. Intra-Sample Correlation (Within Each Sample)

In sample 1, Acceptability_1 shows a strong positive correlation with both Taste_1 and Appearance_1 (r = 0.0 for both), indicating that these attributes significantly influence the overall acceptability of this sample. Consistency_1 exhibits weak or negative correlations with other attributes, such as texturte_1 (r = −0.04) and acceptability_1 (r = 0.16), suggesting that it plays a limited role in driving consumer preference for this sample. Moderate correlations are observed between aroma_1 and texture_1 (r = 0.72), hinting at their combined influence on sensory perception (Figure 6).

In sample 2, acceptability_2 is strongly correlated with appearance_2 (r = 0.68) and taste_2 (r = 0.67), emphasizing their critical role in determining the product’s overall appeal. Consistency_2 demonstrates a higher correlation with other attributes (e.g., texture_2, r = 0.77) compared to sample 1, indicating a strong influence in this sample’s formulation. Attributes like aroma_2 and color_2 display moderate to high correlations with each other (e.g., aroma_2 and color_2, r = 0.55), suggesting interdependencies in sensory evaluation (Figure 6).

In sample 3, Acceptability_3 is moderately correlated with taste_3 (r = 0.60) and appearance_3 (r = 0.58), reinforcing the role of these attributes in overall acceptability. Consistency_3 has a weak or slightly negative correlation with acceptability_3 (r = −0.10), showing a minimal impact on the sample’s appeal (Figure 6).

Strong interdependencies are observed between colur_3 and texture_3 (r = 0.68), indicating that visual and tactile elements influence the sensory perception of this sample (Figure 6).

#### 2.3.2. Inter-Sample Correlation (Across Samples)

Similar attributes across samples, such as taste_1, taste_2, and taste_3, generally exhibit moderate to high correlations, indicating consistent evaluation standards or similarities in product formulation. For example, taste_1 and taste_2 correlate with r = 0.55, showing a moderate level of consistency in taste evaluation between these two samples. Appearance_1 and Appearance_2 show a correlation of r = 0.69, suggesting a relatively high level of similarity in the appearance evaluation. However, variability is observed in attributes like consistency and aroma, where cross-sample correlations are lower. This indicates differences in product characteristics or sensory panel interpretations, highlighting that these attributes may vary significantly between samples (Figure 6).

General Trends across Attributes:

- Appearance and taste consistently demonstrate strong positive correlations with acceptability in all three samples. This indicates that visual appeal and flavor are the primary drivers of consumer preferences.
- Texture and color also show moderate-to-high correlations with acceptability, highlighting their secondary but significant roles in influencing sensory appeal.
- Aroma and consistency generally exhibit a weaker correlation with acceptability, suggesting that they have less influence on consumer preference compared to other attributes.

### 2.4. The Gel with the Best Formulation

The best formulation of the gel is the one that obtained the highest value of the effectiveness index or NH, and the second sample is the best formulation, according to the DeGarmo index. Sample B showed the highest average pH values. However, the average preferences of panelists did not show the highest values in any sensory properties (Table 4).

### 2.5. Shelf Life of Samples

The shelf life of all three samples was observed for 12 days regularly to check if the product quality was maintained or not. Some parameters such as physical appearance, color, texture, and taste were observed, and not much change was observed in the product until the 12th day, and the product was fresh and edible.

According to the statistical analysis of the various parameters of carbohydrate–protein sports gel, it was found that there were no major significant differences between the parameters tested. This is because the ingredients added to the product had approximately the same ratio with a slight difference between all three variations to constrain the nutritional profile of the product for better effectiveness and application.

The market has an availability of gels that consist of carbohydrates, vitamins, and electrolytes for an athlete to use during or pre-workout. Brands like GU Honey Stringer, Maurten gels, GU energy, Clif shot, Huma, etc., consist of different amounts of honey, glucose, fructose, maltodextrin, and sea salt. Many energy gels offer a significant amount of carbohydrates along with other electrolytes that are ideal for endurance athletes [40]. Accel Gel is the only brand that circulates the protein gel in the market along with some percentage of carbohydrates with ingredients of fructose, sucrose, maltodextrin, whey protein, and hydrolysate.

Our proposed gel contains the formulation of a 1:2 ratio of carbohydrates to protein, which makes our gel better than the energy gel available on the market. It has been shown that when athletes consume a gel supplement with a carbohydrate-to-protein ratio with an interval of 30 min, their glycogen synthesis is stimulated at an accelerated speed [41].

The growing contact of individuals with Cannabis sativa has uncovered allergies linked to various components of the plant, particularly hemp seed. However, the primary allergens in hemp seeds have yet to be determined. Numerous known allergen families exist in hemp seeds; particularly notable are the seed storage proteins, vicilins, and edestins as potential hemp seed allergens [42]. Indians most popularly consume pulses, cereals, and vegetables, and food allergy patterns are created by the foods we eat the most. It has been found that chickpea (*Cicer arietinum*) can cause IgE-mediated reactions ranging from rhinitis to anaphylaxis [43]. Extreme allergic responses triggered by honey are uncommon. Honey may contain allergens from pollen proteins (composite plants: ragweed and sagebrush) and protein (enzymes) from Hymenoptera secretion.

Our formulated gel may be an alternative to different supplements for athletes and active individuals, as it is easy to consume and carry and provides an optimal ratio of carbohydrate and protein content mainly targeted toward high-intensity athletes. The formulation has products that have evidence in sports use and have been proven to be beneficial. This may be consumed by a larger number of individuals if they are not prone to the allergens of any of the present substances and are looking for sources to replenish glycogen effectively during the immediate hours of workout or training.

### 2.6. Toxicity Testing

The THC levels in hemp seeds varied between 0.001 and 0.005 mg/g, which is less than the usual range of 5 mg/kg for the total THC content in India (FSSAI). CBD concentrations between 0.040 and 0.068 mg/g are below the average range of 75 mg/kg for total CBD content in India (FSSAI). And the CBN levels are between 0.001 and 0.015 mg/g.

### 2.7. Viscosity Testing

The viscosity measurements revealed that sample C (42 g hemp) exhibited the highest viscosity at 1580 ± 28 cP, followed by sample B (38 g hemp) with a viscosity of 1425 ± 30 cP, and sample A (32 g hemp) with a viscosity of 1350 ± 25 cP. Even with the variations in viscosity, sensory evaluations showed that all samples had a smooth texture and were easy to swallow, as reflected in texture scores ranging from 6.6 to 6.64 and consistency scores between 6.6 and 7. The gels displayed shear-thinning properties, ensuring that they were neither too thick nor too runny, making them suitable for nutritional use in sports.

### 2.8. Texture Profile Analysis

The analysis of the textures in the developed sports gel samples showed variations in hardness, gumminess, and chewiness, which corresponded with the sensory evaluation findings.

- Sample A (32 g of hemp) displayed the least hardness (4.2 ± 0.5 N) and gumminess (3.8 ± 0.4 N), which matched its higher acceptability score (7.16 ± 0.99) due to its soft texture.
- Sample B (38 g of hemp) presented a moderate level of hardness (4.6 ± 0.6 N) and gumminess (4.2 ± 0.5 N), reflecting its texture score of 6.4 ± 1.26 and smooth consistency rating of 6.6 ± 1.41.
- Sample C (42 g of hemp) showed a slightly greater hardness (4.9 ± 0.7 N) and gumminess (4.5 ± 0.6 N) but remained within an easily chewable range, supported by its sensory texture score of 6.6 ± 1.71 and acceptability score of 7 ± 1.19.

The findings show that every formulation preserved a harmonious texture, avoiding excessive firmness while providing sufficient gel consistency suitable for athletes.

## 3. Conclusions

**Figure 7:**
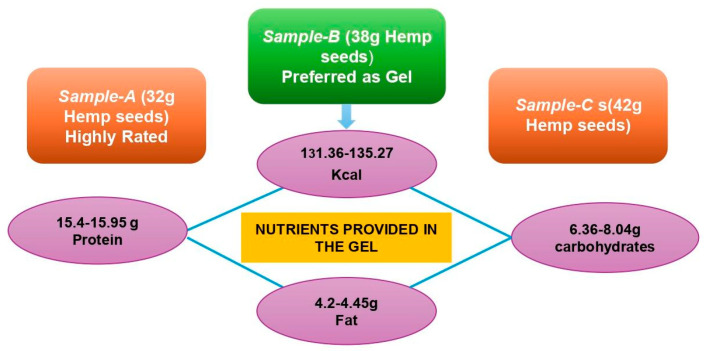
Carbohydrate–protein-rich sports gel nutrition profile (by author Vishal Gill, 2025).

We developed a carbohydrate–protein gel in three variants (A, B, and C) containing different amounts of hemp seeds (32 g, 38 g, and 42 g, respectively). Sample A received the highest ratings across parameters, but sample B was preferred as a gel due to its favorable 1:2 carbohydrate-to-protein ratios, making it an excellent post-workout recovery supplement. The gel provides 131.36–135.27 kcal, 6.36–8.04 g of carbohydrates, 15.4–15.95 g of protein, and 4.2–4.45 g of fat per 50 g serving (Figure 7). It offers a convenient, natural source of macronutrients for athletes, requiring minimal preparation and being easy to carry.

### Limitations

One limitation of this study was that the testing process was carried out in another laboratory that we did not have access to. Using only one gelling agent was another limitation. In this study, the number of subjects taken represented the minimum population of athletes, but the population of athletes varies from sport to sport, so this product might have varied functionality accordingly. Also, the product was prepared during summer, so the shelf life of the product might be different if made in the winter season.

The findings of this study can be used to inform future investigations into sports food formulas appropriate for athletes. Further research can then be conducted with athletes for the efficiency of this product, for further research in comparison with other commercially available gels, and for the long-term effects of this product.

## 4. Materials and Methods

### 4.1. Materials

For the preparation of the gel, we chose the following ingredients: hemp seeds (*Cannabis sativa* L.), Bengal gram dal (*Cicer arietinum* L.), honey, and xanthan gum chosen as a gelling agent, and for flavoring, we added vanilla extract. Hemp seeds were bought from Indian Hemp Seeds and Co., Bengal gram dal was bought from a local shop, honey was bought from Zandu Pure Honey, xanthan gum was bought online (Bake King), and vanilla extract was bought from a local shop. The gels were created using the materials with the compositions shown in Table 5.

### 4.2. Preparation of Sports Carbohydrate–Protein Gels

**Figure 8:**
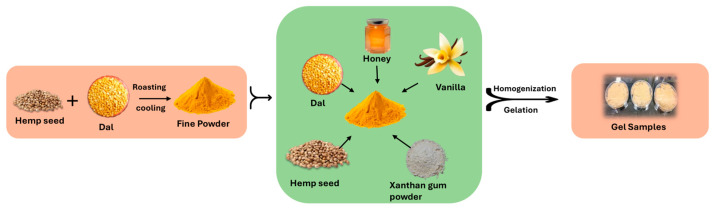
Process of preparing carbohydrate–protein-rich sports gel (by author Vishal Gill, 2025).

**Figure 9:**
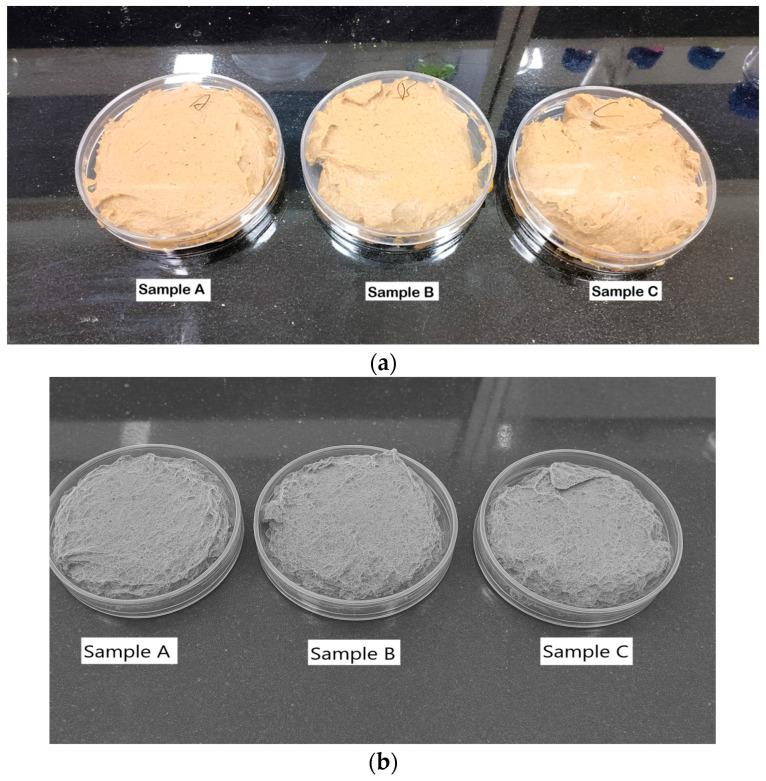
(**a**) Sports gels with different hemp seed concentrations. (**b**) SEM images of sports gels with different hemp seed concentrations (by Vishal Verma, 2024).

A gel from hemp seeds and Bengal gram dal was prepared, which also contained honey and xanthan gum. The gel was made in three variations to obtain different amounts of protein in each product from hemp seeds. The composition of the gel was as follows: 50 g of Bengal gram dal, 32 g, 38 g, and 42 g, of hemp seeds, 40 g of honey, water, and xanthan gum at 0.5% (%w/w). Dal (Bengal gram) and hemp seeds were roasted at 100 degrees Celsius for 10 min until the dal and seeds became brown, then cooled and ground into fine powders with the mortar and pestle. These powders were mixed with water and honey, and we added vanilla extract for flavoring. Xanthan gum (0.5% by weight) was added, and the mixture was homogenized with the help of a hand homogenizer (Biobase, D160, cat.no: 8034111000) for 5 min at 30 degrees Celsius in a narrow glass beaker. Then, we refrigerated the product for 24 h for gelation. The final products, named A, B, and C, contained 32 g, 38 g, and 42 g of hemp seeds, respectively (Figure 8 and Figure 9a,b).

The product’s ingredients ratio was selected according to the protein intake generally required by the athletes, 0.3–0.4 g/kg. Considering the average weight of athletes, the product was made accordingly so that it contained 21–28 g of protein per serving [44]. The nutrition values are referenced from the IFCT table and formulated in the manner that honey was kept at 40 g to match the carbohydrate content according to the protein.

### 4.3. Physicochemical Characteristics Analysis

Among the attributes examined are pH, carbohydrates, protein, fat, moisture, ash, and gross energy. Carbohydrates, protein, fat, moisture, and ash were tested in the FICCI Research and Analysis Centre, New Delhi. The pH was tested in the bioscience lab of the School of Sports Biosciences, Central University of Rajasthan. The pH was measured by using a benchtop pH meter (Labman Scientific Instruments pH meter, Model No LMPH12, Jaipur, India) where the samples were diluted to a 1:5 ratio and mixed properly before measurements.

The pH of all three gels was tested by the benchtop pH meter in the lab of Sports Biosciences of Central University of Rajasthan. Before starting the machine, the pH standardization was checked to make sure the machine was giving accurate readings. We produced two standard pH solutions, one for a pH of 4 and one for a pH of 7, after turning on the pH meter to warm up. We then put the electrode and thermometer into the solution, and as the temperature stabilized, we verified the pH readings of the known standard. After that, we tested the samples. Because the material was semi-solid, processing was required. To create a paste-like consistency, 0.2 mL of distilled water was mixed with one gram of a sample. The thermometer was then dipped into the sample until the temperature stabilized, and an electrode was also dipped into the paste to obtain independent pH meter readings for each of the three samples [45].

### 4.4. Sensory Characteristics Analysis

The sensory testing of the food product was carried out using the hedonic test in which we involved 25 panelists who were semi-trained. They evaluated appearance, color, consistency, aroma, taste, acceptability, and texture. The sensory characteristics were assessed by a 9-point scale (1 = dislike extremely and 9 = like extremely), then transferred into the increasing number scale [43,44,45,46,47,48]. All panelists were instructed to rinse their mouths with water in between each sample tasting during the evaluation.

The subjects we selected for sensory evaluation were selected on specific criteria, including the following: (1) they should be healthy, not colorblind, and alcohol-free; (2) have not smoked for one hour before the sensory test; (3) not be allergic to or abstain from any of the ingredients in the food item that will be examined [49].

### 4.5. Best Treatment Determination

To determine the effective index of the product, the effective index method by DeGarmo was used to choose the best formulation of the product according to the results of the hedonic test. The effective index produces effectiveness by using valence weight (BV) ranges from 0 to 1, relative weight (BN), an effectiveness value (NE), and a result value (NH) [46,50].

$$BV=\frac{the\;parameter\;average}{the\;parameter’s\;highest\;value\;}\;NE=\frac{Treatment\;value-the\;worse\;value}{The\;best\;value-The\;worst\;value}\;BN_{i}=\frac{BV_{i}}{\sum_{i=1}^{\rho }BV_{i}}+\;NH=BN\times NE\;IE=\sum_{i=1}^{P}NH_{ij}\;P=treatment$$

### 4.6. Toxicity Analysis

Hemp seed gel sample preparation: A tared 50 mL conical bottom tube was filled with 1 g of gel sample and 19 mL of 95% ethanol, and the tube was sealed and vortexed for around 30 s. A 1.5 mL snap-cap centrifuge tube filled with 750 μL of 95% ethanol was filled with an aliquot of 250 μL of extract. After a quick vortex, the sealed tube was centrifuged for five minutes at 5000 RCF. Under vacuum, the supernatant was transferred to an amber LC autosampler vial using a 0.22 μm syringe filter. Before analysis, the vial was sealed and kept at 4 °C [51].

Preparation of stock solutions and standards of cannabinoids: Stock solutions of individual cannabinoids were created at concentrations of 100 mg/mL or 10 mg/mL in ethanol by weighing the appropriate amount of the standard into a tared, de-static 4 mL amber vial and adding the necessary volume of ethanol to reach the target concentration. A mixed cannabinoid calibrator solution was formed by combining 0.1 mL from each major cannabinoid solution (CBD, Δ9-THC, and CBN at 10 mg/mL) into a 4 mL amber vial and then diluting with 2.9 mL of ethanol. Cannabinoid spike recovery solutions were made using 10 mg/mL concentrations of Δ9-THC and CBN, along with a 100 mg/mL concentration of CBD. The standards were kept at −20 °C when not being utilized [51].

Spike recovery sample preparation: The gel product was weighed and homogenized before being placed in 50 mL centrifuge tubes. Two fortification levels of cannabinoid standard solutions (10 μg/g and 10 mg/g of CBD; 10 μg/g and 100 μg/g of Δ9-THC and CBN) were added to it. Spike recovery samples were analyzed alongside the product [51].

HPLC/DAD analysis: A modified Agilent 1260 Infinity II LC (Agilent Technologies; Santa Clara, CA, USA) comprising a binary pump, high-performance autosampler with a thermostat, column compartment, and DAD equipped with a 60 mm path length/4 μL flow cell was used to perform the analysis in accordance with Ciolino method. The analysis was conducted at 25 °C with preheating using an ACE Excel 3 C18-AR column. After injecting 2 μL of the sample and washing it for 5 s with 70% ethanol, the autosampler was kept at 8 °C. For 16 min, isocratic conditions of 33% water with 0.5% acetic acid (A) and 67% acetonitrile (B) at 0.3 mL/min were maintained. This was followed by a 1 min ramp to 95% B, a 1 min hold, and a 6 min re-equilibration at 67% B. Absorption at 240 nm and 270 nm was used for quantification, and UV/Vis absorbance was measured at 220 nm, 240 nm, 270 nm, and 307 nm. The measured concentration of an extract was multiplied by the sample dilution factor to determine the amount of cannabinoids present in the products [51,52,53].

### 4.7. Viscosity Analysis

A PCE-RVI 2 viscometer (PCE Instruments, Mumbai, India), featuring a measuring range of 15 to 100,000 cP and an accuracy of <±2% of the measuring range, was used to assess the rheological properties of the developed sports gel, utilizing an L0 spindle configuration with a resolution of 0.01 cP. This evaluation followed the method outlined by Baroyi et al. (2023) [54]. The measurements were performed at a consistent temperature of 25 °C, regulated by a water circulation system. Prior to testing, samples were allowed to acclimate to room temperature for one hour. The shear rate was gradually increased from 0.01 to 100 s^−1^, and the final apparent viscosity was determined as the average of 100 data points.

### 4.8. Texture Profile Analysis

A Texture Profile Analysis (TPA) was conducted utilizing a TA-HD Plus texture analyzer (Stable Micro Systems, UK), adhering to a standardized protocol. A 12.7 mm diameter probe (Delrin P/0.5R) equipped with a 50 kg load cell was employed to assess textural characteristics. The experiment was performed at a consistent speed of 2.0 mm/s during the pre-test, test, and post-test stages, applying 50% compression and a trigger force of 4.793 g as noted by Baroyi et al. (2023) [54]. Data were collected at a rate of 100 points per second, and hardness and gumminess metrics were captured automatically using Texture Expert for Windows 1.20 software.

### 4.9. Data Analysis

The pH data were analyzed using one-way ANOVA and Duncan’s multiple range test with a 95% confidence level to determine mean differences. Sensory attributes were analyzed with the non-parametric Kruskal–Wallis test, followed by Bonferroni comparison using SPSS version 29.0.10. The DeGarmo effectiveness index approach was then used to determine the most effective course of action.

## Figures and Tables

**Table 1:** pH of gel.

Sample No.	pH ± SD
A	5.794 ± 0.016
B	5.822 ± 0.0183
C	5.785 ± 0.015

SD—standard deviation, A—sample A, B—sample B, and C—sample C.

**Table 2:** Lab report on energy, carbohydrates, protein, fat, ash, and moisture of three samples of gels with different variations: A, B, and C with different hemp seed contents of 32 g, 38 g, and 42 g, respectively.

Sample No.	Energy (Kcal)	Carbohydrates (g)	Protein (g)	Fat (g)	Ash (g)	Moisture (g)
A	262.7	12.73	30.9	8.9	1.92	44.65
B	263.2	16.09	30.8	8.4	1.77	42.94
C	270.5	15.71	31.9	8.9	1.85	41.64

g—grams; Kcal—kilocalorie.

**Table 3:** Mean values of sensory characteristics of the gels. Sample A = 32 g of hemp seeds, Sample B = 38 g of hemp seeds, and Sample C = 42 g.

Sensory Characteristics							
Treatment ^1^	Appearances ± SD	Taste ± SD	Color ± SD	Texture ± SD	Aroma ± SD	Consistency ± SD	Acceptability ± SD
Sample A	6.36 ± 1.41	7.04 ± 1.10	6.56 ± 1.29	6.64 ± 1.38	7.68 ± 0.94	7 ± 1.19	7.16 ± 0.99
Sample B	6.28 ± 1.40	6.68 ± 1.44	6.56 ± 1.29	6.4 ± 1.26	7.6 ± 1.16	6.6 ± 1.41	7 ± 1.26
Sample C	6.68 ± 1.11	6.96 ± 1.17	6.48 ± 1.36	6.6 ± 1.71	7.48 ± 1.09	6.96 ± 1.31	7 ± 1.19

^1^ The values are expressed as means ± SD (n = 25), SD—standard deviation.

**Table 4:** Effectiveness index determination.

Variables	BV	BN	Sample A		Sample B		Sample C	
			NE	NH	NE	NH	NE	NH
Appearances	0.805	0.126	0.590	0.073	0.570	0.071	0.560	0.073
Taste	0.766	0.120	0.510	0.062	0.613	0.072	0.592	0.071
Color	0.726	0.113	0.512	0.058	0.593	0.068	0.496	0.055
Texture	0.727	0.114	0.528	0.060	0.567	0.064	0.600	0.068
Aroma	0.843	0.132	0.560	0.074	0.650	0.087	0.620	0.080
Consistency	0.761	0.119	0.500	0.060	0.520	0.060	0.592	0.071
Acceptability	0.784	0.122	0.540	0.066	0.600	0.074	0.500	0.060
pH	0.995	0.155	0.480	0.074	0.533	0.084	0.500	0.077
Total	6.408	1.000		0.527		0.580 ^1^		0.556

^1^ Determination of the best formulation of the gels according to the rank of panelists (n = 25) for all parameters (appearances, taste, color, texture, aroma, consistency, acceptability, pH). BV = weight of valence; BN = relative weight; NE = effectiveness value; NH = result value. The best formulation is shown by the highest total of NH values. Sample A = 32 g hemp seeds, Sample B = 38 g hemp seeds, Sample C = 42 g hemp seeds.

**Table 5:** Ingredients of the gel.

Ingredients (g)	Treatments		
	Sample A	Sample B	Sample C
Bengal gram dal	50	50	50
Hemp seeds	32	38	42
Honey	40	40	40
Water	40	50	60
Xanthan gum	0.82	0.89	0.96

g = grams.

## Data Availability

The original contributions presented in this study are included in the article. Further inquiries can be directed to the corresponding author.
